# Metformin Use Is Associated With Reduced Inflammation and Pain After Total Knee Arthroplasty in Patients With Type 2 Diabetes

**DOI:** 10.1111/os.70352

**Published:** 2026-06-03

**Authors:** Zhongyu Luo, Xiaoying Zhong, Chen Zhang, Mingwei Hu, Muyang Yu, Bin Feng, Xisheng Weng

**Affiliations:** ^1^ Department of Orthopedics Peking Union Medical College Hospital Beijing People's Republic of China; ^2^ State Key Laboratory of Complex Severe and Rare Diseases, Peking Union Medical College Hospital Peking Union Medical College and Chinese Academy of Medical Sciences Beijing People's Republic of China; ^3^ Department of Breast Surgery Peking Union Medical College Hospital Beijing People's Republic of China

**Keywords:** Inflammation, Metformin, Pain, Total knee arthroplasty, Type 2 diabetes mellitus

## Abstract

**Objective:**

Postoperative inflammation and pain remain key barriers to recovery after total knee arthroplasty (TKA). Metformin has anti‐inflammatory properties, but its perioperative impact in TKA is unclear. This study evaluated whether regular metformin use reduces postoperative inflammation and pain in T2DM patients undergoing TKA.

**Methods:**

In this single‐center retrospective cohort study, patients with Type 2 diabetes mellitus (T2DM) who underwent primary TKA between December 2024 and May 2025 were included. Patients were grouped based on whether they had regularly taken metformin. 1:1 propensity score matching was performed to reduce baseline differences between groups, yielding 60 matched pairs. Inflammatory markers including C‐reactive protein (CRP), interleukin‐6 (IL‐6), erythrocyte sedimentation rate (ESR), and D‐dimer were measured preoperatively and on postoperative days 1 and 3. Pain was assessed using the Visual Analogue Scale (VAS) on postoperative days 1 and 3. Linear mixed‐effects models were used to compare outcomes between groups after adjusting for residual confounders.

**Results:**

In the matched cohort, the metformin group showed significantly lower CRP levels on postoperative day 3 (107.04 ± 49.19 vs. 130.91 ± 59.62 mg/L, *p* = 0.02), lower IL‐6 levels on postoperative day 1 (62.22 ± 47.17 vs. 91.07 ± 41.55 pg/mL, *p* < 0.01), and postoperative day 3 (31.33 ± 20.84 vs. 57.74 ± 59.98 pg/mL, *p* < 0.01), and lower ESR levels on postoperative day 3 (45.40 ± 17.85 vs. 47.05 ± 20.19 mm/h, *p* = 0.03) than the non‐metformin group. VAS pain scores on postoperative day 3 were also significantly lower in the metformin group (3.03 ± 1.03 vs. 4.15 ± 1.25, *p* < 0.01). These differences remained significant after adjusting for confounders.

**Conclusion:**

Long‐term regular use of metformin is associated with reduced acute inflammation and postoperative pain in T2DM patients undergoing TKA. As a widely used and well‐tolerated medication, metformin holds promise as a low‐cost, adjunctive therapeutic strategy to enhance early recovery and improve perioperative outcomes in diabetic patients undergoing TKA.

## Graphical abstract

Long‐term metformin use in T2DM patients undergoing TKA significantly reduced postoperative inflammation and pain. On postoperative day 3, CRP, IL‐6, ESR, D‐dimer levels and VAS scores were markedly lower in the metformin group, suggesting potential perioperative anti‐inflammatory and analgesic benefits.

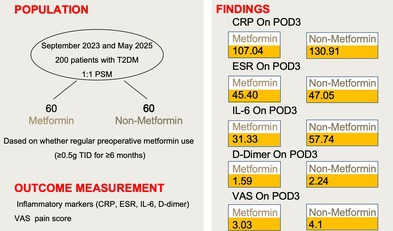

**List of Abbreviations**
AMPK: AMP‐activated protein kinase
BMI: body mass index
CRP: C‐reactive protein
ESR: erythrocyte sedimentation rate
IL‐6: Interleukin‐6
KOA: knee osteoarthritis
NF‐κB: nuclear factor kappa B
RA: rheumatoid arthritis
SD: standard deviation
T2DM: Type 2 diabetes mellitus
TKA: total knee arthroplasty
VAS: visual analogue scale

## 1 Introduction

Total knee arthroplasty (TKA) is the gold‐standard treatment for end‐stage knee osteoarthritis (KOA) and one of the most successful orthopedic interventions in recent decades [1, 2, 3]. TKA significantly alleviates pain, improves joint function, and enhances quality of life. With global population aging and rising obesity rates, the demand for TKA is expected to continue increasing [1, 4, 5]. However, postoperative inflammation and pain remain critical factors affecting early rehabilitation [6, 7, 8]. The postoperative inflammatory cascade can lead to joint swelling, fibrin deposition, and periprosthetic tissue damage, while acute pain may progress to chronic pain and central sensitization, further hindering recovery. Effective perioperative strategies to mitigate inflammation and pain are therefore of paramount clinical importance.

KOA and Type 2 diabetes mellitus (T2DM) are increasingly co‐occurring, with studies showing that approximately 21%–25% of TKA recipients also have T2DM [9, 10, 11]. These patients often experience heightened inflammatory responses, with elevated serum C‐reactive protein (CRP), interleukin‐6 (IL‐6), and erythrocyte sedimentation rate (ESR) levels compared to non‐diabetic individuals [12]. Moreover, T2DM is associated with increased postoperative pain and delayed functional recovery [13, 14], potentially due to hyperglycemia‐induced oxidative stress, proinflammatory cytokine release, and insulin resistance [15].

Metformin is the first‐line pharmacologic therapy for T2DM and has garnered attention for its pleiotropic effects beyond glycemic control. Mechanistically, metformin activates AMP‐activated protein kinase (AMPK), suppresses NF‐κB signaling, downregulates proinflammatory cytokines, and modulates macrophage polarization toward an anti‐inflammatory M2 phenotype. Previous studies have reported its potential role in reducing osteoarthritis progression, lowering the risk of arthroplasty [16, 17]. However, no previous study has specifically investigated its perioperative effects on acute postoperative inflammation and pain after TKA.

This study aims to evaluate (i) whether regular metformin use is associated with reduced postoperative inflammatory responses after TKA; (ii) whether metformin use is associated with reduced postoperative pain. We hypothesize that metformin reduces perioperative inflammation and improves early recovery outcomes through its anti‐inflammatory mechanisms.

## 2 Methods

### 2.1 Study Design and Ethical Approval

This was a single‐center retrospective cohort study of patients with T2DM who underwent primary TKA between December 2024 and May 2025. The study was approved by the Institutional Review Board (No. K6954), and the requirement for written informed consent was waived because of the retrospective study design.

### 2.2 Patient Selection

Patients were eligible if they were 18–80 years of age, had symptomatic primary or secondary knee osteoarthritis requiring primary TKA, and had a documented diagnosis of T2DM. Patients were excluded if they had infectious arthritis, rheumatoid arthritis, bilateral TKA during the same hospital admission, perioperative urinary tract infection, perioperative respiratory infection, or postoperative body temperature > 39.0°C.

### 2.3 Data Collection and Grouping

Demographic and clinical data were extracted retrospectively from the electronic medical record system. Baseline variables included age, sex, body mass index (BMI), glycated hemoglobin (HbA1c), surgical approach, and preoperative hemoglobin (HGB) and hematocrit (Hct). Surgical approach was categorized as traditional or robot‐assisted.

Perioperative inflammatory markers, including CRP, ESR, IL‐6, and D‐dimer, were recorded preoperatively, on postoperative day 1, and on postoperative day 3. Postoperative pain was assessed using the visual analogue scale (VAS) on postoperative days 1 and 3.

To evaluate perioperative blood loss, HGB and Hct values measured preoperatively and on postoperative day 3 were collected. The changes in HGB and Hct were calculated as follows: ΔHGB = preoperative HGB − postoperative HGB, and ΔHct = preoperative Hct − postoperative Hct.

Patients were classified into the metformin group if they had regularly used metformin preoperatively at a dose of at least 0.5 g three times daily for at least 6 months before surgery. Patients who did not meet this definition were assigned to the non‐metformin group. Perioperative glucose management was standardized. Oral hypoglycemic agents were discontinued on the day of surgery and temporary insulin therapy was used for glycemic control. After oral intake was resumed on postoperative day 1, patients were switched back to their preoperative oral antidiabetic medications.

### 2.4 Propensity Score Matching

To reduce baseline differences between groups, propensity score matching (PSM) was performed using a logistic regression model in which regular metformin use was the dependent variable. The propensity score was estimated based on sex, age, BMI, HbA1c, and surgical approach. Patients in the metformin and non‐metformin groups were matched in a 1:1 ratio using nearest‐neighbor matching. Covariate balance before and after matching was assessed using standardized mean differences (SMDs). An absolute SMD < 0.1 was considered indicative of adequate balance.

### 2.5 Statistical Analysis

All statistical analyses were performed using R software (version 4.3.3). Continuous variables are presented as mean ± standard deviation, and categorical variables as number (%). Between‐group comparisons of continuous variables were performed using Student's *t*‐test or the Mann–Whitney U test, as appropriate, while categorical variables were compared using the chi‐square test or Fisher's exact test. Baseline characteristics before and after matching were summarized descriptively, and covariate balance was assessed using SMDs.

In the propensity score‐matched cohort, perioperative inflammatory markers, pain scores, and blood loss‐related indicators were first compared descriptively between groups. To further account for repeated measurements within individuals over time, linear mixed‐effects models were fitted in the matched cohort for CRP, ESR, IL‐6, D‐dimer, and VAS. Fixed effects included group, time, and the group‐by‐time interaction, and a patient‐level random intercept was included to account for within‐subject correlation. Given the small residual imbalances after matching, age, BMI, and HbA1c were further adjusted for in the models. Adjusted means, adjusted mean differences, 95% confidence intervals (CIs), and *p*‐values were reported for each outcome at each time point. A two‐sided *p*‐value < 0.05 was considered statistically significant.

## 3 Results

**FIGURE 1:**
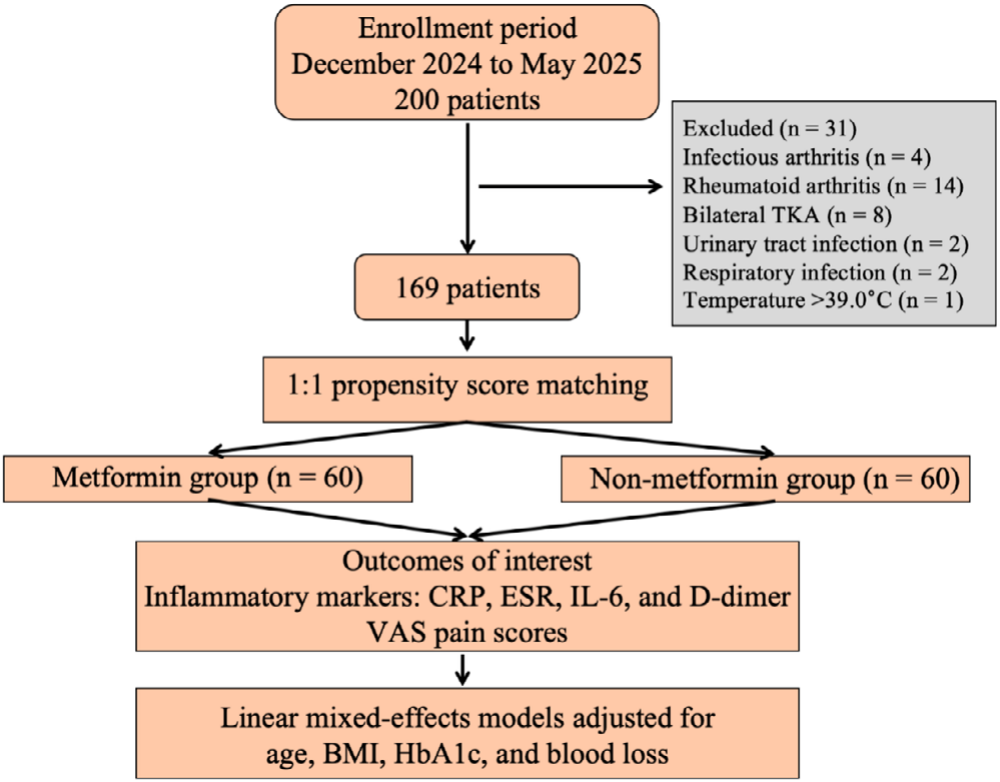
Flow diagram of patient enrollment and group allocation. A total of 200 patients who underwent primary total knee arthroplasty during the enrollment period from December 2024 to May 2025 were screened. Thirty‐one patients were excluded, including those with infectious arthritis (*n* = 4), rheumatoid arthritis (*n* = 14), bilateral total knee arthroplasty (*n* = 8), urinary tract infection (*n* = 2), respiratory infection (*n* = 2), and temperature > 39.0°C (*n* = 1). The final cohort included 169 patients. Patients were then divided into two groups based on whether they had used metformin regularly (≥ 0.5 g TID for ≥ 6 months) before surgery: the metformin group (*n* = 106) and the non‐metformin group (*n* = 63). After 1:1 propensity score matching, 60 patients were included in the metformin group and 60 in the non‐metformin group.

A total of 200 patients with T2DM who underwent primary TKA between December 2024 and May 2025 were initially screened. Thirty‐one patients were excluded, including those with infectious arthritis (*n* = 4), rheumatoid arthritis (*n* = 14), bilateral TKA during the same hospital admission (*n* = 8), urinary tract infection (*n* = 2), respiratory infection (*n* = 2), and postoperative body temperature > 39.0°C (*n* = 1). Ultimately, 169 patients were included in the final cohort, including 106 in the metformin group and 63 in the non‐metformin group (Figure 1).

After 1:1 propensity score matching, 60 matched pairs were generated. Baseline covariate balance improved after matching, although small residual imbalances remained in age, BMI, and HbA1c (Table 1).

**TABLE 1:** Baseline characteristics before and after propensity score matching.

Variables	Before matching			After matching		
	Non‐metformin (*n* = 63)	Metformin (*n* = 106)	SMD	Non‐metformin (*n* = 60)	Metformin (*n* = 60)	SMD
Sex, Male, *n* (%)	17 (27.0)	20 (18.9)	0.194	14 (23.3)	12 (20.0)	0.081
Age, years	69.02 ± 5.94	68.27 ± 6.81	0.116	68.95 ± 6.02	67.92 ± 7.34	0.154
BMI, kg/m^2^	27.09 ± 3.91	26.70 ± 4.04	0.096	26.73 ± 3.63	27.11 ± 3.17	0.114
HbA1c, %	6.88 ± 1.13	6.75 ± 0.98	0.123	6.75 ± 0.86	6.88 ± 1.11	0.130
Traditional approach, *n* (%)	49 (77.8)	77 (72.6)	0.119	46 (76.7)	45 (75.0)	0.039

*Note:* Values are presented as mean ± standard deviation for continuous variables and number (%) for categorical variables. Propensity scores were estimated using sex, age, BMI, HbA1c, and surgical approach, followed by 1:1 nearest‐neighbor matching. Covariate balance was assessed using standardized mean differences (SMDs).

### 3.1 Inflammatory Markers

**FIGURE 2:**
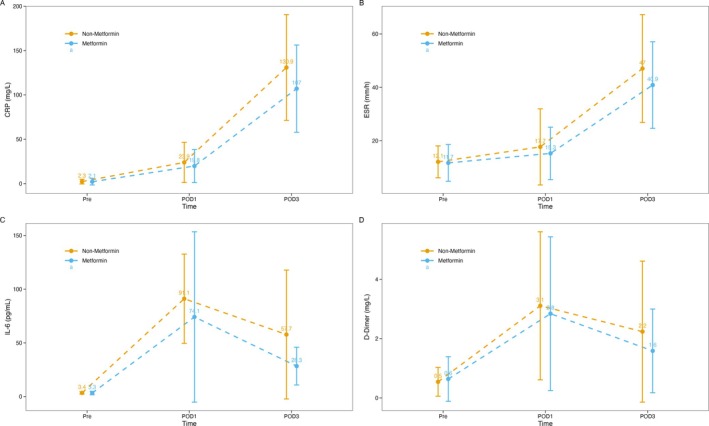
Perioperative changes in inflammatory markers between metformin and non‐metformin groups. (A) C‐reactive protein (CRP), (B) erythrocyte sedimentation rate (ESR), (C) interleukin‐6 (IL‐6), and (D) D‐dimer levels were recorded at three timepoints: Preoperatively (Pre), postoperative day 1 (POD1), and postoperative day 3 (POD3). Data are shown as mean ± standard deviation.

In the propensity score‐matched cohort, perioperative inflammatory markers and pain scores were compared between the two groups (Table 2, Figure 2). On postoperative day 3, CRP levels were lower in the metformin group than in the non‐metformin group (107.04 ± 49.19 vs. 130.91 ± 59.62 mg/L, *p* = 0.02). ESR levels on postoperative day 3 were also lower in the metformin group (45.40 ± 17.85 vs. 47.05 ± 20.19 mm/h, *p* = 0.03). IL‐6 levels were markedly lower in the metformin group on postoperative day 1 (62.22 ± 47.17 vs. 91.07 ± 41.55 pg/mL, *p* < 0.01) and postoperative day 3 (31.33 ± 20.84 vs. 57.74 ± 59.98 pg/mL, *p* < 0.01). D‐dimer levels on postoperative day 3 were lower in the metformin group (1.59 ± 1.41 vs. 2.24 ± 2.38 mg/L, *p* = 0.02). No clear between‐group differences were observed in preoperative values, postoperative day 1 CRP, ESR, and D‐dimer. The mean ΔHGB was 24.6 ± 8.7 g/L in the non‐metformin group and 23.9 ± 8.2 g/L in the metformin group (*p* = 0.64), while the mean ΔHct was 7.4% ± 2.6% and 7.1% ± 2.4%, respectively (*p* = 0.51). No significant between‐group differences were observed in these blood loss‐related indicators.

**TABLE 2:** Perioperative inflammatory markers and pain scores in TKA patients with and without metformin use.

Variables	Time points	Non‐metformin (*n* = 60)	Metformin (*n* = 60)	*p*
CRP, mg/L	Pre	2.27 ± 2.66	2.10 ± 3.66	0.27
	POD1	23.89 ± 22.69	19.80 ± 18.68	0.70
	POD3	130.91 ± 59.62	107.04 ± 49.19	0.02
ESR, mm/h	Pre	12.12 ± 5.97	12.57 ± 8.14	0.51
	POD1	17.71 ± 14.26	16.92 ± 11.44	0.42
	POD3	47.05 ± 20.19	45.40 ± 17.85	0.03
IL‐6, pg/mL	Pre	3.39 ± 1.15	3.17 ± 1.41	0.29
	POD1	91.07 ± 41.55	62.22 ± 47.17	< 0.01
	POD3	57.74 ± 59.98	31.33 ± 20.84	< 0.01
D‐dimer, mg/L	Pre	0.54 ± 0.48	0.64 ± 0.75	0.98
	POD1	3.11 ± 2.49	2.84 ± 2.59	0.28
	POD3	2.24 ± 2.38	1.59 ± 1.41	0.02
VAS score	POD1	3.08 ± 1.87	2.86 ± 1.81	0.81
	POD3	4.15 ± 1.25	3.03 ± 1.03	< 0.01

*Note:* Values are presented as mean ± standard deviation.

Abbreviations: CRP = C‐reactive protein; ESR = erythrocyte sedimentation rate; IL‐6 = interleukin‐6; POD1 = postoperative day 1; POD3 = postoperative day 3; PRE = preoperative; VAS = Visual Analogue Scale for pain.

After adjustment for residual imbalances, CRP remained significantly lower in the metformin group on postoperative day 3 (*p* < 0.01). ESR was also significantly lower in the metformin group on postoperative day 3 (*p* = 0.01). Similarly, IL‐6 levels were significantly lower in the metformin group on postoperative day 1 (*p* = 0.03) and postoperative day 3 (*p* < 0.01) (Table 3).

**TABLE 3:** Adjusted perioperative inflammatory markers and pain scores in the propensity score‐matched cohort using linear mixed‐effects models.

Variables	Time points	Non‐metformin	Metformin	*p*
CRP, mg/L	Pre	2.42 (−6.17 to 11.01)	1.93 (−6.65 to 10.51)	0.94
	POD1	24.04 (15.45 to 32.63)	19.63 (11.05 to 28.21)	0.48
	POD3	131.06 (122.47 to 139.65)	106.87 (98.29 to 115.45)	< 0.01
ESR, mm/h	Pre	12.13 (8.75 to 15.50)	11.46 (8.09 to 14.82)	0.78
	POD1	17.72 (14.34 to 21.10)	15.02 (11.65 to 18.39)	0.27
	POD3	47.06 (43.68 to 50.43)	40.60 (37.23 to 43.97)	0.01
IL‐6, pg/mL	Pre	3.81 (−7.24 to 14.86)	2.87 (−8.18 to 13.92)	0.91
	POD1	91.49 (80.44 to 102.54)	73.68 (62.64 to 84.73)	0.03
	POD3	58.17 (47.12 to 69.21)	27.92 (16.87 to 38.97)	< 0.01
D‐dimer, mg/L	Pre	0.54 (0.06 to 1.01)	0.68 (0.20 to 1.15)	0.68
	POD1	3.10 (2.62 to 3.58)	2.88 (2.40 to 3.36)	0.52
	POD3	2.23 (1.75 to 2.71)	1.62 (1.14 to 2.10)	0.08
VAS score	POD1	3.08 (2.68 to 3.48)	2.85 (2.46 to 3.25)	0.42
	POD3	4.15 (3.75 to 4.55)	3.00 (2.60 to 3.40)	< 0.01

*Note:* Linear mixed‐effects models were fitted in the propensity score‐matched cohort, with fixed effects for group, time, and the group‐by‐time interaction, and a patient‐level random intercept. Given the small residual imbalances after matching, age, BMI, HbA1c, and blood loss were further adjusted for in the models. Data are presented as mean (standard deviation) with corresponding 95% confidence intervals (CI).

Abbreviations: CRP = C‐reactive protein; ESR = erythrocyte sedimentation rate; IL‐6 = interleukin‐6; POD1 = postoperative day 1; POD3 = postoperative day 3; PRE = preoperative; VAS = Visual Analogue Scale.

### 3.2 Pain Scores

On postoperative day 3, the metformin group also demonstrated significantly lower VAS pain scores compared to the non‐metformin group (3.03 ± 1.03 vs. 4.15 ± 1.25, *p* < 0.01) (Table 2), and this difference remained significant after adjustment for residual imbalances (Table 3). No statistically significant difference in VAS scores was observed between the groups on postoperative day 1.

To further assess the clinical relevance of the observed biochemical differences, additional postoperative clinical outcomes were compared between groups. NSAID use was comparable between the non‐metformin and metformin groups (96.7% vs. 95.0%, *p* = 0.61), whereas opioid use was lower in the metformin group (40.0% vs. 23.3%, *p* = 0.04). Postoperative fever rate (41.6% vs. 36.7%, *p* = 0.78) and length of stay (8.7 ± 1.2 vs. 8.4 ± 1.3 days, *p* = 0.67) were similar between the two groups.

## 4 Discussion

### 4.1 Metformin Was Associated With Reduced Acute Postoperative Inflammatory Responses After TKA

This study demonstrated that long‐term regular metformin use was associated with reduced acute postoperative inflammatory responses in T2DM patients undergoing TKA. Compared with the non‐metformin group, patients receiving metformin showed significantly lower CRP, IL‐6, and ESR levels during the early postoperative period, particularly on postoperative day 3. These findings suggest that metformin may confer perioperative anti‐inflammatory benefits in patients undergoing TKA.

The perioperative anti‐inflammatory and analgesic effects of metformin can likely be attributed to its well‐characterized multifaceted anti‐inflammatory mechanisms. Metformin activates the AMPK pathway, which in turn suppresses proinflammatory signaling cascades such as NF‐κB. This leads to reduced expression and release of key cytokines [18]. In addition, metformin modulates immune cell function, particularly by promoting macrophage polarization from the proinflammatory M1 phenotype to the anti‐inflammatory M2 phenotype [19]. These combined mechanisms provide a plausible explanation for the reduced CRP and IL‐6 levels and alleviated postoperative pain observed in the metformin group. Moreover, the decrease in D‐dimer levels may indicate attenuated activation of the coagulation‐fibrinolysis system, suggesting reduced tissue injury and inflammatory burden.

Interestingly, the between‐group differences became more apparent on postoperative day 3 rather than postoperative day 1. This finding may indicate that metformin primarily modulates the sustained postoperative inflammatory response instead of the immediate surgical stress reaction induced by tissue trauma during surgery. From a clinical perspective, excessive postoperative inflammation may impair early mobilization, increase postoperative discomfort, and potentially delay rehabilitation after TKA. Therefore, attenuation of postoperative inflammatory responses may contribute to improved perioperative recovery.

### 4.2 Metformin Was Associated With Reduced Postoperative Pain After TKA

In addition to the reduced inflammatory response, metformin use was also associated with lower postoperative pain scores. In the present study, VAS scores on postoperative day 3 were significantly lower in the metformin group, accompanied by lower postoperative opioid use. These findings suggest that the anti‐inflammatory effects of metformin may also translate into clinically meaningful analgesic benefits during the early postoperative recovery period.

Postoperative pain after TKA is closely associated with inflammatory mediator release, peripheral sensitization, and tissue edema. Therefore, attenuation of systemic inflammation may partially explain the lower pain scores observed in metformin users. Previous experimental studies have also suggested that metformin may exert direct analgesic effects through modulation of AMPK‐related signaling pathways and suppression of neuroinflammatory responses. Although the exact mechanisms remain incompletely understood, our findings support the hypothesis that metformin may contribute to both anti‐inflammatory and analgesic effects during the perioperative period.

### 4.3 Comparison With Previous Studies

Our findings align with previous research on the anti‐inflammatory properties of metformin across a range of diseases and postoperative scenarios. In chronic inflammatory conditions such as rheumatoid arthritis, metformin has demonstrated beneficial effects: randomized controlled trials have shown that adding metformin to conventional therapy significantly reduces systemic inflammation, decreases disease activity, and improves quality of life [20]. In degenerative joint diseases like osteoarthritis, there is growing evidence supporting metformin's anti‐inflammatory and analgesic effects. A recent randomized clinical trial in overweight or obese patients with KOA reported that oral metformin administration significantly alleviated joint pain and improved physical function and daily activity levels [21]. Moreover, large‐scale epidemiological studies suggest a potential protective role of metformin against joint degeneration. A cohort study involving over 40,000 patients found that consistent metformin use in individuals with T2DM was associated with an approximately 30% reduced risk of undergoing total knee or hip arthroplasty [22]. These findings imply that metformin may delay disease progression by mitigating chronic inflammation and metabolic dysfunction. In the surgical context, although few studies have specifically addressed acute postoperative inflammation in TKA, some evidence supports metformin's positive impact on surgical recovery. For instance, a retrospective matched cohort study reported a lower incidence of postoperative complications—particularly periprosthetic joint infection and deep vein thrombosis—in diabetic patients receiving metformin [23]. Collectively, these findings—from chronic disease prevention to improved surgical recovery—underscore metformin's broad anti‐inflammatory potential and clinical relevance. However, it is noteworthy that clinical studies specifically targeting the acute postoperative phase in TKA remain scarce. Our study addresses this gap by focusing on short‐term perioperative outcomes, offering novel insights and valuable clinical implications.

## 5 Limitations

Despite its strengths, this study has several limitations. As a single‐center retrospective cohort study, it cannot fully eliminate residual confounding despite adjustments. The patient population was limited to T2DM individuals from one geographic region, which may affect generalizability. Additionally, our findings apply specifically to diabetic patients, and the effects of metformin in non‐diabetic populations remain unknown. Finally, the study focused only on short‐term outcomes within 3 days postoperatively, without long‐term follow‐up on recovery or joint function. From a clinical perspective, our findings suggest that metformin may have potential value in perioperative glucose‐lowering strategies for diabetic patients undergoing TKA, particularly considering its associations with reduced postoperative inflammation and pain. Nevertheless, given the retrospective design and inherent limitations of the present study, these results are not sufficient to support a definitive change in clinical practice. Further prospective randomized controlled studies are needed to confirm this potential benefit.

## 6 Conclusion

Long‐term regular use of metformin in T2DM patients undergoing TKA is associated with reduced acute postoperative inflammation and lower pain scores. As a widely used and well‐tolerated medication, metformin holds promise as a low‐cost, adjunctive therapeutic strategy to enhance early recovery and improve perioperative outcomes in diabetic patients undergoing TKA.

## Author Contributions

**Zhongyu Luo:** conceptualization, data curation, formal analysis, writing – original draft, methodology, visualization. **Muyang Yu:** data curation, validation. **Xiaoying Zhong+:** data curation, validation, formal analysis, conceptualization. **Chen Zhang:** data curation, formal analysis, writing – original draft. **Mingwei Hu:** data curation, validation. **Bin Feng:** conceptualization, methodology, supervision, resources, writing – review and editing. **Xisheng Weng:** conceptualization, methodology, resources, supervision, writing – review and editing, funding acquisition.

## Funding

This study was supported by the National Natural Science Foundation of China (No. 82572715).

## Disclosure

All authors listed meet the authorship criteria according to the latest guidelines of the International Committee of Medical Journal Editors; All authors are in agreement with the manuscript.

## Ethics Statement

This study was approved by the Institutional Review Board (No: K6954). The requirement for written informed consent was waived due to the retrospective nature of the study.

## Conflicts of Interest

The authors declare no conflicts of interest.

## Data Availability

The data that support the findings of this study are available from the corresponding author upon reasonable request.
